# Common mental disorders in pregnancy and postnatal depressive symptoms in the MINA-Brazil study: occurrence and associated factors

**DOI:** 10.11606/s1518-8787.2022056004028

**Published:** 2022-09-16

**Authors:** Bruno Pereira da Silva, Alicia Matijasevich, Maíra Barreto Malta, Paulo A R Neves, Maria Cristina Mazzaia, Maria Cristina Gabrielloni, Márcia C Castro, Marly Augusto Cardoso

**Affiliations:** I Universidade Federal do Acre Cruzeiro do Sul AC Brasil Universidade Federal do Acre. Campus Floresta. Cruzeiro do Sul, AC, Brasil; II Universidade de São Paulo Faculdade de Medicina Departamento de Medicina Preventiva São Paulo SP Brasil Universidade de São Paulo. Faculdade de Medicina. Departamento de Medicina Preventiva. São Paulo, SP, Brasil; III Universidade de São Paulo Faculdade de Saúde Pública Departamento de Nutrição São Paulo SP Brasil Universidade de São Paulo. Faculdade de Saúde Pública. Departamento de Nutrição. São Paulo, SP, Brasil; IV Universidade Católica de Santos Programa de Pós-Graduação em Saúde Coletiva Santos SP Brasil Universidade Católica de Santos. Programa de Pós-Graduação em Saúde Coletiva. Santos, SP, Brasil; V Universidade Federal de Pelotas Programa de Pós-Graduação em Epidemiologia Pelotas RS Brasil Universidade Federal de Pelotas. Programa de Pós-Graduação em Epidemiologia. Pelotas, RS, Brasil; VI Universidade Federal de São Paulo Escola Paulista de Enfermagem Departamento de Enfermagem Clínica e Cirúrgica São Paulo SP Brasil Universidade Federal de São Paulo. Escola Paulista de Enfermagem. Departamento de Enfermagem Clínica e Cirúrgica. São Paulo, SP, Brasil; VII Universidade Federal de São Paulo Escola Paulista de Enfermagem Departamento de Enfermagem na Saúde da Mulher São Paulo SP Brasil Universidade Federal de São Paulo. Escola Paulista de Enfermagem. Departamento de Enfermagem na Saúde da Mulher. São Paulo, SP, Brasil; VIII Harvard T.H. Chan School of Public Health Department of Global Health and Population Boston MA United States of America Harvard T.H. Chan School of Public Health. Department of Global Health and Population. Boston, MA, United States of America

**Keywords:** Pregnant Women, Mental Disorders, epidemiology, Depression, Postpartum, Risk Factors, Cohort Studies

## Abstract

**OBJECTIVE:**

To investigate the occurrence and factors associated with common mental disorders in pregnancy and depressive symptoms in postpartum, as well as the association between both in the Brazilian Western Amazon.

**METHODS:**

This is a prospective cohort in the MINA-Brazil study with women who received primary health care in the town of Cruzeiro do Sul, Acre State. We performed two clinical evaluations during pregnancy (the first: 16–20 weeks; the second: 28 gestational weeks) and three postpartum evaluations (at 3, 6 and 12 months), in which demographic and socioeconomic, gestational, lifestyle and clinical data were collected. We used the Self-Reported Questionnaire (score ≥ 8) to screen the gestational common mental disorder and the Edinburgh Postnatal Depression Scale (score ≥ 10) to identify postpartum depressive symptoms. We used adjusted ordinal logistic regression to investigate the relationship between the covariates and the occurrence of common mental disorders in pregnancy and postpartum depressive symptomatology.

**RESULTS:**

A total of 461 women completed the two clinical evaluations in pregnancy; of these, 247 completed the three postpartum evaluations. The occurrence of common mental disorder during pregnancy was 36.2% and 24.5% in the first and second evaluations, respectively, and the cumulative incidence was 9.2%. In addition, 50.3% maintained the disorder between evaluations. During postpartum, approximately 20% of the mothers presented depressive symptoms during the first year of their children’s lives. Parity (≥ 2) was associated with common mental disorders, while low maternal education was associated with postpartum depressive symptoms. Women with a common mental disorder in both evaluations during pregnancy were 5.6 times more likely (95%CI: 2.50–12.60) to develop postpartum depressive symptoms.

**CONCLUSION:**

The occurrence of common mental disorder at any time assessed during pregnancy, but especially its persistence from the second trimester, was strongly associated with depressive symptoms after childbirth. These findings highlight the need for early screening and monitoring of the mental health of pregnant women at the start of prenatal care in order to reduce possible negative impacts on the health of the mother-child binomial caused by such events.

## INTRODUCTION

During the perinatal period, which goes from the beginning of pregnancy to 12 months postpartum, women are subject to some types of mental disorders^1^, of which the common mental disorders (CMD) are among the most prevalent. CMD are a suspected mental comorbidity for mood, anxiety and somatization disorders. Their characteristics involve depressive and anxious symptomatology, deconcentration, forgetfulness, insomnia, fatigue, irritability and non-specific somatic complaints, which can cause consternation and dysfunction in daily activities^2^. The postpartum period presents greater susceptibility to the occurrence of non-psychotic depressive symptoms with a dysphoric mood, psychomotor disorder, sleep and appetite changes, fatigue, feelings of worthlessness or excessive guilt, recurrent thoughts of death, with suicidal ideation, feelings of inadequacy and rejection of the baby^2^.

Recent systematic reviews show high rates of gestational CMD in the world: in high-income countries, prevalence rates are estimated at between 10%–15%, while in low-and middle-income countries, rates are estimated at between 10%–41%^3 , 4^. Regarding postpartum depressive symptomatology, estimated prevalence rates vary between 24%–27% and 14%–50% in high-income and low-and middle-income countries, respectively^4^. In Brazil, studies show alarming prevalence rates of CMD in pregnancy (63%) and postpartum depressive symptoms (26%)^5^.

Several biological, psychological and social factors have been pointed out as potential determinants of such mental disorders in the perinatal period^6^. In this sense, some risk factors are particularly relevant in some cultures and in low-and middle-income countries, which helps explain the great magnitude of mental disorders during pregnancy and postpartum in certain places. Some of those factors include teenage pregnancy, unplanned or unwanted pregnancy, nulliparity, low socioeconomic status, household overcrowding and physical, psychological and verbal violence perpetrated by the partner^6 , 7^. In the postpartum period, depressive and anxious symptoms in pregnancy, a previous history of mental disorder, lack of family support, living without a partner, being a single mother and multiparity are commonly associated with mental illness^7 , 8^. In addition, the occurrence of CMD in pregnancy has been pointed out as an important predictor for the emergence of depressive symptoms in the postpartum period^3^.

The occurrence of mental disorders in the perinatal period has profound negative impacts for both the mother-child binomial and the entire family and society. Unfavorable obstetric and neonatal outcomes – such as obstetric complications and negative childbirth experiences, prematurity, low birth weight and impaired mother-infant interaction – have been pointed out as consequences of mental disorders in the perinatal period, in addition to family problems resulting from the emergence or aggravation of marital conflicts, for example^6 , 9^.

Despite the importance and impact that mental disorders occurring in the perinatal period have on the affected woman’s family and social life, the understanding of this phenomenon among women in low and middle-income countries is still limited, therefore it requires investigation to establish preventive and health-promoting strategies^3 , 4^.

This study seeks to investigate the frequency and factors associated with CMD and depressive symptoms in the postpartum period, as well as the association between both in a cohort of pregnant women who received prenatal care in primary health services in a town in Brazil’s Western Amazon. Our hypothesis is that the occurrence of gestational CMD is a risk factor for the emergence of postnatal depressive symptoms.

## METHODS

### Study Design, Location and Studied Population

This is a prospective cohort study that is part of the MINA-Brazil Project (Maternal and Child Health and Nutrition in Cruzeiro do Sul, Acre)^10^, which investigates health and nutrition determinants of the mother-child binomial from the prenatal period to the child’s first two years of life in Cruzeiro do Sul, Acre State. This is the second largest town in the state, with approximately 82,000 inhabitants, 70% of which live in the urban area. It is located 640 km from the state capital, Rio Branco^11^. The town’s Human Development Index in 2010 was 0.664^11^. Only 12.7% of households had access to adequate sanitation in 2010^11 , 12^.

To participate in the study, we considered women up to 20 weeks into pregnancy, since the date of their last menstruation, who lived in the urban area, received prenatal care in one of the town’s 13 primary health care facilities and planned to give birth in the town’s only maternity hospital. Only women with a singleton pregnancy and low obstetric risk were included in this analysis^10 , 13^.

The participants were monitored weekly between February 2015 and January 2016. During this period, Family Health Strategy teams recorded all contact information of potential participants in a standardized manner. Subsequently, telephone contact was made to explain the research protocol and the to invite the potential participants.

Once they had accepted, the research team made home visits to collect the participants’ signatures for the Informed Consent Form (ICF) and to apply the sociodemographic and health history questionnaires^10 , 13^. Then, the first evaluation was scheduled to take place between 16 and 20 gestational weeks for collection of clinical data, blood samples, additional health history and lifestyle information, as well as an ultrasound to confirm gestational age. A second clinical evaluation took place when the women were around their 28^th^ gestational week. Both evaluations took place between March 2015 and May 2016^10 , 13^.

Between July 2015 and June 2016, the participants gave birth in the town’s only maternity hospital. In the 3^rd^ postpartum week, a telephone interview was conducted between August 2015 and September 2016 at the *Laboratório de Pesquisas Telefônicas da Faculdade de Saúde Pública* , Universidade de São Paulo. The following evaluations took place 6 months (January and December 2016) and 12 months (August 2016 and July 2017) after childbirth in face-to-face evaluations. In each of these three postpartum evaluations, we collected information on maternal mental health^10^. More details about the steps are shown in the Figure .

**Figura:**
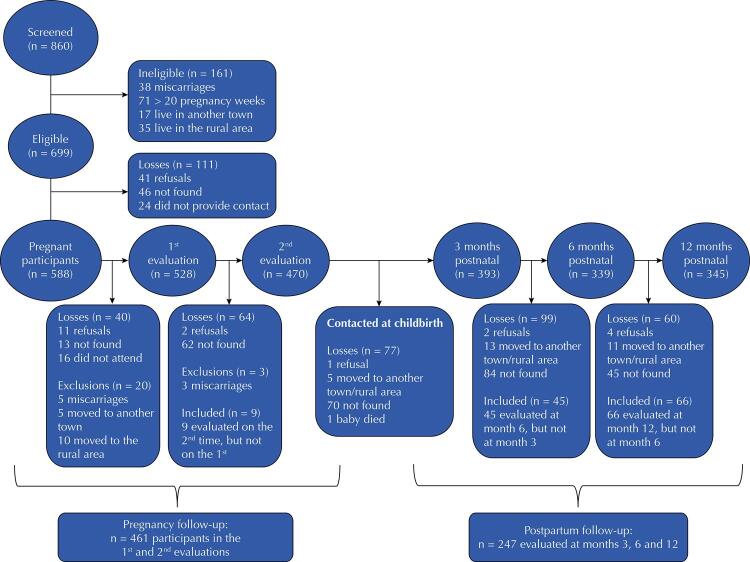
Flowchart of recruitment and gestational and postnatal follow-ups of women participating in the MINA-Brasil study.

### Exposure and Outcome

For the present analysis, we regarded as outcomes of interest the occurrence of CMD in the first and second evaluations, performed during pregnancy, and depressive symptoms in the postpartum period at months 3, 6 and 12.

To monitor the exposure of interest characterized by depressive symptoms, anxiety and some psychosomatic complaints in the last 30 days, we used the Brazilian version of the Self-Reported Questionnaire (SRQ-20), validated in 1986 by Mari and Williams^14^, which has an 0.86 internal consistency index (Cronbach’s alpha). The SRQ-20 has 20 questions with yes/no answers, and each positive answer equals one point. The higher the frequency of positive answer, the greater the intensity of CMD. The cutoff point adopted to classify CMD in pregnancy was ≥ 8 (75.4% sensitivity and 90.9% specificity)^15^.

To evaluate depressive symptoms in the postnatal period, we used the Edinburgh Postnatal Depression Scale (EPDS), which estimates the occurrence of depressive symptoms in the last seven days^16^. The version used in this study was translated and validated by Santos et al.^17^, who achieved a 0.87 internal consistency index (Cronbach’s alpha). The questionnaire is structured with ten questions and four possible answers each, with the score in each question ranging from 0 to 3 and the total score from 0 to 30 points. The higher the frequency of positive responses, the more intense the depressive symptomatology, with the cutoff point of ≥ 10 being adopted for this analysis (82.7% sensitivity and 65.3% specificity)^17^.

### Covariates

We divided the continuous or discrete covariates into three groups: demographic and socioeconomic; gestational; lifestyle and clinical. Demographic and socioeconomic variables were: maternal age in years (≤ 19, 20–34, ≥ 35), maternal schooling in years (0–9, 10–11, ≥ 12), self-reported skin color (white, black, brown, indigenous or yellow), living with a partner (no/yes), head of family (mother, partner, other person), household overcrowding (≥ 2 people per room at home, no/yes), *Bolsa Família* recipient (no/yes), having consumer goods at home.

To assess the socioeconomic status of the study participants, we calculated the wealth index based on possession of consumer goods and household appliances. The goods index was obtained by analyzing the main components, later divided into quintiles, with the first quintile representing the poorest 20% and the highest quintile the richest 20%. The adoption of this method is justified by its capacity to portray the family’s economic status better than just considering the monthly income of family members, which is inaccurate and difficult to obtain^10^.

The gestational variables were parity (0, 1, ≥ 2), planned pregnancy (no/yes); lifestyle: smoking (no/yes) and alcohol consumption (no/yes); clinical evaluation: anemia in pregnancy (no/yes), pre-pregnancy body mass index (BMI) (underweight, adequate weight, overweight, obesity). Anemia in pregnancy was determined based on the concentration of blood hemoglobin (Hb) measured with the portable Hemocue^®^ hemoglobinometer (Ängelholm, Sweden) with venous blood. To assess anemia in pregnant women, we adopted the classification of < 110g/L at sea level, recommended by the World Health Organization (WHO)^18^. Pre-pregnancy weight self-reported by the pregnant women was collected in the first clinical evaluation, considering a period up to 2 months before pregnancy^19^. The participants’ height was measured using a portable vertical stadiometer (exact height, Belo Horizonte, Brazil) with accuracy of 0.1 cm and capacity of 213 cm. For the anthropometric assessment of pregnant women aged 19 years or older, we used the pre-pregnancy BMI, which is calculated by dividing pre-pregnancy weight in kg by height in meters squared (BMI = pre-pregnancy weight/height^2^), and classified according to WHO criteria^19^: low weight (BMI < 18.5 kg/m^2^); adequate weight (BMI = 18.5–24.9 kg/m^2^); overweight (BMI = 25.0–29.9 kg/m^2^) and obesity (≥ 30 kg/m^2^). For pregnant women under 19 years of age, nutritional status was assessed with the aid of the WHO Anthro Plus application (http://www.who.int/growthref/tools/en/), which calculates BMI for age in z-score, considering the following cutoff points for assessment: underweight (z-score < -2); adequate weight (z-score -2 ˫ +1); overweight (z-score ≥ +1) and obesity (z-score ≥ +2).

### Statistical Analysis

Measures of central tendency and dispersion were calculated and used in the sample description (mean, median and standard deviation [SD]), as well as the respective 95% confidence intervals (95%CI). With the frequency rates of CMD in each of the evaluations, we determined the cumulative incidence (proportion of pregnant women with a positive SRQ-20 in the second evaluation among those with a negative result in the first evaluation) and the persistence of CMD (proportion of women with a positive result in the second evaluation among those with a positive result in the first evaluation) between the first and second evaluation. We verified the existence of possible differences between participants who were not followed and those who completed the evaluations during pregnancy and postpartum period by comparing the means of maternal age, wealth index and years of education using the *t* test.

To determine the factors associated with CMD outcomes in pregnancy and postpartum depressive symptoms separately, we adopted the hierarchical covariate selection model proposed by Victora et al.^20^ For this analysis, the covariates were grouped into the following levels, from the farthest to the closest: socioeconomic; gestational and obstetric; and clinical. The adjusted analysis took into account the effect of each variable controlling for confounding variables at the same level and at higher levels. The adjusted analysis included only variables with a p-value < 0.20 at each level.

We used adjusted ordinal logistic regression to investigate the relationship between the covariates and the occurrence of CMD in pregnancy and postpartum depressive symptomatology. For this, the information collected in the different periods during pregnancy and postpartum were combined according to the persistence of each symptomatology. Regarding CMD, women with SRQ-20 < 8 points in both evaluations in pregnancy were classified as “never had CMD”, women with SRQ-20 ≥ 8 in at least one evaluation were classified as “once” and participants with SRQ-20 ≥ 8 in both evaluations were classified as “always had CMD”. A similar approach was adopted for postpartum depressive symptoms, based on the score defined for this study (EPDS ≥ 10), which classifies women with depressive symptoms as “never”, “once” or “always” after the birth of the baby. Ordinal logistic regression assumes that the exposure effect is the same for all category divisions of the outcome variable. Data analyses were performed using the Stata 14.0 statistical package.

### Ethical Aspects

The MINA-Brasil project was approved by the Research Ethics Committee of the Faculdade de Saúde Pública, Universidade de São Paulo (Opinion No. 872.613, November 13, 2014). This analysis was approved by the Research Ethics Committee of the Universidade Federal de São Paulo (Opinion No. 2,726,516, 06/20/2018). Participation in the study was voluntary and the participants signed the ICF.

## RESULTS

We screened 860 pregnant women throughout the study period, of which 699 (81.3%) were eligible to participate in the study. Of these, 588 answered the socioeconomic questionnaire. A total of 528 participants (75.5% of the eligible women) completed the first clinical evaluation, at pregnancy weeks 16 and 20, and 470 (67.2% of eligible) completed the second evaluation at pregnancy week 28. A total of 461 pregnant women attended the two clinical evaluations. In the postpartum period, 393, 339 and 345 women answered the EPDS at 3, 6 and 12 months, respectively. We included in the longitudinal analysis ( Figure ) 247 participants who answered the EPDS at all postpartum moments.

In the comparison of the socioeconomic characteristics between the participants followed during all stages of the study and the follow-up losses, we did not find differences between the mean ages (CMD: 24.9 and 23.9 years; p = 0.152; depressive symptom: 25.2 and 24.3 years; p = 0.076) and years of maternal schooling (CMD: 10.5 and 9.9 years; p = 0.055; depressive symptom: 10.6 and 10.2 years; p = 0.076). We only found differences for the wealth index, in which women who participated in the clinical evaluations both during pregnancy and postpartum period had a higher mean index compared to those who did not complete the evaluations (CMD: 1.8 and 1.6; p = 0.001; depressive symptom: 1.9 and 1.7; p = 0.008).

The occurrence of CMD in the first and second pregnancy evaluations was 36.2% (95%CI: 32.1–40.4) and 24.5% (95%CI: 20.6–28.6), respectively. The cumulative incidence of CMD was 9.2% (95%CI: 6.1–13.1) and symptom persistence occurred in half of the pregnant women, 50.3% (95%CI: 42.5–58.1). In the postnatal period, we observed prevalence rates for symptom persistence of 22% (95%CI: 18.6–27.1) at month 3, 20.9% (95%CI: 16.7–25.7) at month 6 and 20.6% (95%CI: 16.4–25.2) at month 12 after childbirth (data not shown in table).

Table 1 presents the socioeconomic, gestational and obstetric and clinical characteristics of the participants. Most of the participants were between 20–34 years old (65%), about 30% had less than 9 years of education and 40% were beneficiaries of *Bolsa Família* . Less than 15% of the women were the head of household, the vast majority lived with a partner (77.6%) and 44% were in their first pregnancy ( Table 1 ). The mean gestational age in the first evaluation was 20 weeks (SD = 2.91) and 27.7 weeks (SD = 1.61) in the second evaluation (data not shown in tables).

**Table 1:** Characteristics of the participants and prevalence, incidence and persistence of common mental disorders in pregnancy. Cruzeiro do Sul, Acre, 2015–2017.

Variables	16^th^ to 20^th^ gestational week			28^th^ gestational week			Incidence		Persistence		Common mental disorders between 1^st^ and 2^nd^ evaluations				
				
	n (%)	Prevalence (%)	p	n (%)	Prevalence (%)	p	%	p	%	p	n (%)	Never	Once	Always	p
Wealth index (quintiles)			0.201			0.263		0.817		0.163					0.227
1	95 (18.0)	41.1		79 (16.8)	29.1		4.4		61.3		76 (16.5)	56.6	18.4	25.0	
2	110 (20.9)	42.7		95 (20.2)	30.5		9.1		59.0		94 (20.4)	53.2	22.3	24.5	
3	106 (20.1)	34.9		98 (20.9)	24.5		9.7		52.9		96 (20.8)	58.3	22.9	18.8	
4	109 (20.7)	33.0		101 (21.5)	19.8		10.8		38.2		99 (21.5)	58.6	28.3	13.1	
5	107 (20.3)	29.0		97 (20.6)	19.6		10.5		37.9		96 (20.8)	62.5	26.0	11.5	
Head of household			0.565			0.075		0.313		0.107					0.103
Mother	72 (13.7)	41.7		65 (13.8)	35.4		10.8		66.7		64 (13.9)	51.6	20.3	28.1	
Partner	278 (52.8)	35.3		248 (52.8)	21.8		6.7		50.6		245 (53.1)	60.8	21.6	17.6	
Other	177 (33.6)	35.0		157 (33.4)	24.2		12.4		41.8		152 (33.0)	55.9	29.0	15.1	
Recipient of *Bolsa Família*			0.588			0.154		0.227		0.574					0.356
No	316 (60.0)	35.1		288 (61.3)	22.2		7.6		48.5		281 (61.0)	60.5	22.8	16.7	
Yes	211 (40.0)	37.4		182 (38.7)	28.0		11.8		52.9		180 (39.0)	53.9	25.6	20.6	
Household overcrowding (≥ 2 people per room)			0.078			0.069		0.988		0.275					0.047
No	469 (89.0)	34.8		425 (90.4)	23.3		9.2		48.6		416 (90.2)	59.4	23.8	16.8	
Yes	58 (11.0)	46.6		45 (9.6)	35.6		9.1		60.9		45 (9.8)	44.4	24.4	31.1	
Maternal education (years)			0.087			0.107		0.412		0.580					0.329
0–9	142 (27.9)	42.3		126 (27.8)	28.6		8.6		52.9		121 (27.1)	52.9	24.8	22.3	
10–11	242 (47.5)	34.3		213 (46.9)	25.8		11.6		52.8		210 (47.1)	58.1	23.8	18.1	
≥ 12	125 (24.6)	29.6		115 (25.3)	17.4		6.3		42.9		115 (25.8)	65.2	21.7	13.0	
Maternal age (years)			0.450			0.951		0.241		0.352					0.418
≤ 19	143 (27.1)	32.2		123 (26.2)	23.6		13.6		40.5		118 (25.6)	59.3	28.0	12.7	
20–34	343 (65.1)	37.0		309 (65.7)	24.9		7.9		53.9		306 (66.4)	57.5	22.2	20.3	
≥ 35	41 (7.8)	41.5		38 (8.1)	23.7		4.6		46.7		37 (8.0)	56.8	24.3	18.9	
Lives with partner			0.160			0.263		0.501		0.393					0.345
No	118 (22.4)	41.5		101 (21.5)	28.7		6.9		56.1		99 (21.5)	54.6	22.2	23.2	
Yes	409 (77.6)	34.5		369 (78.5)	23.3		9.8		48.4		361 (78.5)	58.8	24.3	16.9	
Skin color			0.549			0.639		0.826		0.571					0.655
White	76 (14.4)	38.2		70 (14.9)	20.0		4.8		42.9		70 (15.1)	57.1	25.7	17.1	
Black	23 (4.4)	39.1		20 (4.3)	30.0		7.7		71.4		20 (4.3)	60.0	15.0	25.0	
Brown	405 (76.9)	35.1		363 (77.2)	24.8		10.0		51.2		354 (76.8)	58.8	23.5	17.8	
Indigenous	4 (0.8)	75.0		4 (0.9)	50.0		0		66.7		4 (0.9)	25.0	25.0	50.0	
Yellow	19 (3.6)	36.8		13 (2.8)	23.1		14.3		33.3		13 (2.8)	46.2	38.5	15.4	
Planned pregnancy			0.007			0.283		0.913		0.921					0.097
No	291 (55.3)	41.2		257 (54.8)	26.5		9.4		50.0		253 (55.0)	53.4	26.1	20.6	
Yes	235 (44.7)	29.8		212 (45.2)	22.2		9.1		50.8		207 (45.0)	63.3	21.3	15.5	
Parity			< 0.001			0.027		0.674		0.205					0.005
0	234 (44.4)	29.1		210 (44.7)	22.9		10.6		49.2		202 (43.8)	62.4	22.8	14.9	
1	134 (25.4)	31.3		120 (25.5)	18.3		8.5		39.5		120 (26.0)	62.5	25.0	12.5	
≥ 2	159 (30.2)	50.3		140 (29.8)	32.1		7.0		57.4		139 (30.2)	47.5	24.5	28.1	
Pre-pregnancy BMI			0.482			0.905		0.822		0.539					0.626
Low weight	38 (7.2)	44.7		35 (7.5)	28.6		5.0		69.2		33 (7.2)	57.6	15.2	27.3	
Adequate weight	325 (61.6)	35.4		287 (61.1)	24.4		10.0		48.1		284 (61.6)	57.0	25.4	17.6	
Overweight	128 (24.2)	33.6		114 (24.3)	22.8		9.3		48.6		110 (23.9)	61.8	22.7	15.5	
Obesity	37 (7.0)	43.2		34 (7.2)	26.5		5.3		53.3		34 (7.4)	52.9	23.5	23.5	
Anemia in pregnancy in the 1^st^ evaluation			0.965			0.547		0.482		0.128					0.248
No	437 (84.9)	36.2		384 (84.4)	23.4		9.8		47.5		384 (84.4)	57.6	25.3	17.2	
Yes	78 (15.1)	35.9		71 (15.6)	26.8		6.5		64.0		71 (15.6)	60.6	16.9	22.5	
Smoking in the 1^st^ evaluation			0.006			0.024		0.431		0.124					0.007
No	508 (96.2)	35.0		444 (96.3)	23.2		0		48.7		444 (96.3)	58.8	24.1	17.1	
Yes	20 (3.8)	65.0		17 (3.7)	47.1		9.4		72.7		17 (3.7)	35.3	17.7	47.1	
Alcohol consumption in the 1^st^ evaluation			0.003			0.060		0.206		0.161					0.010
No	486 (92.1)	34.4		426 (92.4)	23.0		9.7		48.3		426 (92.4)	59.2	24.2	16.7	
Yes	42 (7.9)	57.1		35 (7.6)	37.1		0		65.0		35 (7.6)	42.9	20.0	37.1	

BMI: body mass index.

In the crude analysis, higher frequency of CMD (“always”) during pregnancy ( Table 1 ) was associated with women with household overcrowding, women with parity ≥ 2 and women who smoked and/or consumed alcohol during pregnancy. Table 2 presents the final adjusted model of factors associated with CMD at some point during pregnancy. Only parity ≥ 2 was significantly associated with this outcome, with an odds ratio of 1.72 (95%CI: 1.12–2.66).

**Table 2:** Factors associated with the occurrence of common mental disorders between the first and second clinical evaluations in pregnancy. Cruzeiro do Sul, Acre, 2015–2017.

Variables	_crude_OR (95%CI)	_adjusted_OR (95%CI)	p
Household overcrowding (≥ 2 people per room)			0.109
No	Reference	Reference	
Yes	1.49 (0.86–2.57)	1.62 (0.87–2.94)	
Planned pregnancy			0.101
No	1.56 (1.10–2.21)	1.35 (0.93–1.96)	
Yes	Reference	Reference	
Parity			0.013
0	Reference	Reference	
1	1.05 (0.67–1.62)	0.96 (0.61–1.53)	
≥ 2	2.11 (1.40–3.17)	1.72 (1.12–2.66)	
Smoking in the 1^st^ evaluation			0.144
No	Reference	Reference	
Yes	2.74 (1.08–7.00)	2.06 (0.78–5.47)	
Alcohol consumption in the 1^st^ evaluation			0.052
No	Reference	Reference	
Yes	2.01 (1.06–3.80)	1.95 (0.99–3.83)	

OR: odds ratio; 95%CI: 95% confidence interval.

Table 3 shows the crude analysis for the occurrence of depressive symptoms in the postpartum period. Depressive symptoms during pregnancy were more frequent “always” in women from families with a lower wealth index, women whose mother was the head of the family, women with lower education, with parity ≥ 2, women who had gestational anemia at the first evaluation, and those who consumed alcohol during pregnancy ( Table 3 ). Women who presented a positive SRQ in both the first and second evaluations had the highest frequency rates of depressive symptoms in the postpartum period. In the adjusted analysis for potential confounders, less educated women and those with a history of CMD during pregnancy had the highest chances of postpartum depressive symptomatology ( Table 4 ). Having less than 12 years of schooling increased the chances of postpartum depression by more than three times compared to women with 12 or more years of schooling. Pregnant women who reported always having CMD during pregnancy were 5.6 times more likely (95%CI: 2.50–12.60) to have postpartum depressive symptoms compared to those who never reported the problem ( Table 4 ).

**Table 3:** Variables associated with the occurrence of depressive symptomatology between postnatal months 3, 6 and 12. Cruzeiro do Sul, Acre, 2015-2017 (n = 247).

Variables	n (%)	Depressive symptomatology between months 3, 6 and 12			p

		Never	Once	Always	
Wealth index (quintiles)					0.001
1	35 (14.2)	45.7	25.7	28.6	
2	53 (21.5)	62.3	30.2	7.6	
3	47 (19.0)	61.7	27.7	10.6	
4	59 (23.9)	69.5	28.8	1.7	
5	53 (21.5)	79.3	17.0	3.8	
Head of household					0.040
Mother	29 (11.7)	58.6	17.2	24.1	
Partner	139 (56.3)	64.8	28.1	7.2	
Someone else	79 (32.0)	68.4	25.3	6.3	
Recipient of *Bolsa Família*					0.197
No	153 (61.9)	69.3	23.5	7.2	
Yes	94 (38.1)	58.5	29.8	11.7	
Household overcrowding (≥ 2 people per room)					0.057
No	237 (96.0)	65.8	26.2	8.0	
Yes	10 (4.1)	50.0	20.0	30.0	
Maternal education (years)					0.002
0–9	54 (22.8)	53.7	31.5	14.8	
10–11	118 (49.8)	59.3	31.4	9.3	
≥ 12	65 (27.4)	84.6	13.9	1.5	
Maternal age (years)					0.720
≤ 19	62 (25.1)	72.6	21.0	6.5	
20–34	167 (67.6)	62.9	27.5	9.6	
≥ 35	18 (7.3)	61.1	27.8	11.1	
Lives with partner					0.123
No	54 (21.9)	66.7	18.5	14.8	
Yes	193 (78.1)	64.8	28.0	7.3	
Skin color					0.443
White	30 (12.2)	60.0	23.3	16.7	
Black	11 (4.5)	36.7	45.5	18.2	
Brown	195 (79.0)	67.2	25.1	7.7	
Indigenous	4 (1.6)	75.0	25.0	0	
Yellow	7 (2.8)	71.4	28.6	0	
Planned pregnancy					0.155
No	133 (53.9)	61.7	26.3	12.0	
Yes	114 (46.2)	69.3	25.4	5.3	
Parity					0.044
0	116 (47.0)	70.7	23.3	6.0	
1	59 (23.9)	71.2	22.0	6.8	
≥ 2	72 (29.1)	51.4	33.3	15.3	
Pre-pregnancy BMI					0.259
Low weight	18 (7.6)	44.4	33.3	22.2	
Adequate weight	140 (58.8)	69.3	24.3	6.4	
Overweight	66 (27.7)	60.6	30.3	9.1	
Obesity	14 (5.9)	71.4	21.4	7.1	
Anemia in pregnancy in the 1^st^ evaluation					0.026
No	200 (81.0)	69.0	22.5	8.5	
Yes	47 (19.0)	48.9	40.4	10.6	
Smoking in the 1^st^ evaluation					0.391
No	237 (96.0)	65.8	25.7	8.4	
Yes	10 (4.0)	50.0	30.0	20.0	
Alcohol consumption in the 1^st^ evaluation					0.004
No	226 (91.5)	66.8	26.1	7.1	
Yes	21 (8.5)	47.6	23.8	28.6	
Positive SRQ in the 1^st^ evaluation					< 0.001
No	157 (66.5)	73.9	24.2	1.9	
Yes	79 (33.5)	46.8	31.7	21.5	
Positive SRQ in the 2^nd^ evaluation					< 0.001
No	168 (76.4)	70.8	25.0	4.2	
Yes	52 (23.6)	44.2	34.6	21.2	
Positive SRQ at some point during pregnancy					< 0.001
Never	130 (59.6)	73.1	24.6	2.3	
In an evaluation	49 (22.5)	63.3	28.6	8.2	
Always	39 (17.9)	35.9	35.9	28.2	

BMI: body mass index; SRQ: Self-Reported Questionnaire.

**Table 4:** Factors associated with depressive symptomatology between postnatal months 3, 6 and 12. Cruzeiro do Sul, Acre, 2015–2017.

Variables	_crude_OR (95%CI)	_adjusted_OR (95%CI)	p
Wealth index (quintiles)			0.200
1	5.87 (2.30–14.95)	3.09 (1.15–8.31)	
2	2.27 (0.96–5.33)	1.56 (0.62–3.92)	
3	2.43 (1.01–5.84)	1.96 (0.79–4.90)	
4	1.57 (0.67–3.69)	1.33 (0.55–3.23)	
5	Reference	Reference	
Maternal education (years)			0.007
0–9	5.29 (1.83–15.27)	3.89 (1.58–9.62)	
10–11	3.56 (1.31– 9.66)	3.16 (1.44–6.93)	
≥ 12	Reference	Reference	
Anemia in pregnancy			0.075
No	Reference	Reference	
Yes	0.48 (0.26–0.88)	1.87 (0.94–3.72)	
Alcohol consumption in pregnancy			0.081
No	Reference	Reference	
Yes	2.77 (1.09–7.01)	2.61 (0.89–7.65)	
Positive SRQ at some point during pregnancy			< 0.001
Never	Reference	Reference	
In an evaluation	1.65 (0.83–3.29)	1.87 (0.90–3.92)	
Always	6.19 (2.95–12.97)	5.6 (2.50–12.60)	

OR: odds ratio; 95%CI: 95% confidence interval; SRQ: Self-Reported Questionnaire.

## DISCUSSION

In this prospective study with pregnant women who received prenatal care in primary health services in a town in the Brazil’s Western Amazon, CMD during pregnancy occurred in more than 35% of women in the second trimester and in about a quarter of participants in the third trimester, with approximately 20% of mothers presenting postpartum depressive symptoms during the first year of life of their children. It was also observed that women who presented CMD in the second and third trimesters were almost six times more likely to develop postpartum depressive symptoms compared to women who did not report gestational CMD. Women with two or more children were more likely to suffer CMD during pregnancy and those with lower education were more likely to present depressive symptoms in the postnatal period.

The frequency of CMD at different times of pregnancy in our study was higher than that described in a meta-analysis that included more than 5,700 participants from low-and middle-income countries, 15.6% (95%CI: 15.4–15.9), as well as in an observational study conducted in Ethiopia (26.2%) screened with the SRQ-20 score of ≥ 6^3 , 21^. In Brazil, a cross-sectional study found a high incidence of CMD in the second trimester of pregnancy (51.7%; 95%CI: 51.7–62.6)^22^, while a prevalence rate of 43.1% (95%CI: 40.2–46.1) for CMD in the third trimester was described in Pernambuco^23^. They used the SRQ-20 in the two studies with scores ≥ 7 and ≥ 8, respectively.

Different estimates of postpartum depressive symptomatology were also observed in relation to our findings. A systematic review including more than 38,000 postpartum women found a prevalence of 19.7% (95%CI: 16.9–22.8) of depression^4^. In a cohort including 378 Vietnamese women, the authors described a decrease in the prevalence of postpartum depressive symptomatology (~ 12% in the fourth and sixth months) compared to the pregnancy period (40% and 28% in the first and third pregnancy trimesters, respectively)^24^. A population-based hospital study called “ *Nascer no Brasil* ” showed prevalence rates of 26% of maternal depression, using the EPDS with a score ≥ 13, between 6 hours and 18 months after childbirth in the country. However, maternal depression was more pronounced among women in Brazil’s North region (31.7%)^25^.

Several factors influence the different estimates for mental disorders occurring during pregnancy and in the postpartum period worldwide. However, the criteria adopted to define a case of mental disorder are among those with the biggest influence on the discrepancies found. Although the most widely used instruments for screening these problems in Brazil, and in the international literature, are the SRQ-20 and the EPDS, the cutoff points adopted diverged significantly between the studies, which explains the variability between our results and the literature on the subject^5^. In addition, some studies adopt concepts of CMD that diverge from those established by WHO^2 , 5^.

Several economic and social factors have been associated with the occurrence of mental disorders in the perinatal period, with low maternal education and poor socioeconomic conditions among the most commonly cited^2 , 8 , 26^. Cross-sectional studies conducted in low and middle-income countries indicate that the lack of social support from the family, especially the lack of a partner during pregnancy, are important factors associated with the occurrence of CMD during pregnancy, as well as unplanned pregnancy^21^. Many of these risk factors for mental illness during pregnancy also influence its occurrence in the postpartum period. Several prospective and cross-sectional studies conducted in different countries indicate that unplanned and teenage pregnancy, a history of previous mental illness, lack of social support during pregnancy and episodes of physical, sexual, and/or psychological violence perpetrated by a partner are strongly associated with postpartum depressive symptoms^24 , 25 , 27^.

The occurrence of mental disorders during pregnancy has been investigated as an important risk factor for postpartum mental illness. As shown by findings of a Canadian cohort study with 3,057 participants, the occurrence of at least one risk factor for impaired prenatal mental health (a history of depression treatment, prenatal depressive and anxious symptomatology and adverse psychological experiences during childhood) increased the risk of maternal depression at four months (EPDS ≥ 10) by 2.52 times, while among women who reported all adverse factors, the risk increased by 7.64 times^25^.

Prenatal mental and emotional vulnerability tends to persist after childbirth, causing symptoms already present during pregnancy to transfer into the period when the child needs great maternal attention and care. The literature points out that such perinatal disorders at different times share many of the risk factors associated with their occurrence. These factors tend to last throughout this phase, causing persistence of disorder and mental instability during the course of pregnancy and postpartum period^8 , 25^.

Our findings stress the importance of early detection of mental disorders during pregnancy so that appropriate care can be administered in a timely manner, thus reducing the risks of continuity of such symptoms after childbirth and the possible associated adverse health events^6 , 9^. The SRQ-20 and EPDS are important instruments for quick and easy application and interpretation. They are low cost and do not require a specialist for application. These instruments can be incorporated into the routine of primary health care from the early screening of such conditions, still in the prenatal period, to the postpartum period^16 , 17^.

The strengths of our analysis include its longitudinal design with follow-up of the participants from the prenatal period to 12 months after childbirth, the use of methods validated in Brazil for screening of mental disorders in the perinatal period, and confirmation of gestational age through ultrasound examinations. Limitations include the loss of follow-up during follow-up, mainly due to the difficulty to locate the participants caused by common restrictions in the study area, such as unstable internet connection, lack of adequate direction sings on the streets and intermittent cell phone signal. The possibility of unknown confounders not being considered is important given the observational nature of our study, such as having suffered abuse (violence perpetrated by a romantic partner, sexual violence, physical or sexual abuse in childhood), pressure to have a child and food insecurity.

Among women who received prenatal care from primary health services in Cruzeiro do Sul, Acre State, we found that CMD are common in the second and third trimesters. Also, one in five women reported postnatal depressive symptoms. The occurrence of CMD at any of the evaluated moments during pregnancy, but particularly their persistence from the second trimester on, was strongly associated with mental illness after childbirth. These findings highlight the need for screening and monitoring of mental health for these pregnant women by including an early evaluation of such disorders, still in the prenatal period, to reduce possible negative impacts on the health of the mother-child binomial caused by such events.
