# Supplementary material for: Common mental disorders in pregnancy and postnatal depressive symptoms in the MINA-Brazil study: occurrence and associated factors
Source: Rev Saude Publica. 2022 Sep 16;56:83. doi: 10.11606/s1518-8787.2022056004028 (PMC9529209; doi:10.11606/s1518-8787.2022056004028)
Supplement: Translation

# Translation: Transtorno mental comum na gravidez e sintomas depressivos pós-natal no estudo MINA-Brasil: ocorrência e fatores associados

**Authors:** Bruno Pereira da Silva, Alicia Matijasevich, Maíra Barreto Malta, Paulo A R Neves, Maria Cristina Mazzaia, Maria Cristina Gabrielloni, Márcia C Castro, Marly Augusto Cardoso

**Affiliations:** I Brasil Universidade Federal do Acre. Campus Floresta. Cruzeiro do Sul, AC, Brasil; II Brasil Universidade de São Paulo. Faculdade de Medicina. Departamento de Medicina Preventiva. São Paulo, SP, Brasil; III Brasil Universidade de São Paulo. Faculdade de Saúde Pública. Departamento de Nutrição. São Paulo, SP, Brasil; IV Brasil Universidade Católica de Santos. Programa de Pós-Graduação em Saúde Coletiva. Santos, SP, Brasil; V Brasil Universidade Federal de Pelotas. Programa de Pós-Graduação em Epidemiologia. Pelotas, RS, Brasil; VI Brasil Universidade Federal de São Paulo. Escola Paulista de Enfermagem. Departamento de Enfermagem Clínica e Cirúrgica. São Paulo, SP, Brasil.; VII Brasil Universidade Federal de São Paulo. Escola Paulista de Enfermagem. Departamento de Enfermagem na Saúde da Mulher. São Paulo, SP, Brasil.; VIII United States of America Harvard T.H. Chan School of Public Health. Department of Global Health and Population. Boston, MA, United States of America

**Keywords:** Gestantes, Transtornos Mentais, epidemiologia, Depressão Pós-Parto, Fatores de Risco, Estudos de Coortes

## Abstract

**OBJETIVO:**

Investigar a ocorrência e os fatores associados com os transtornos mentais comuns na gestação e sintomas depressivos no pós-parto, bem como a associação entre ambos na Amazônia Ocidental Brasileira.

**MÉTODOS:**

Coorte prospectiva no estudo MINA-Brasil com mulheres atendidas na atenção primária à saúde de Cruzeiro do Sul, Acre. Foram realizadas duas avaliações clínicas na gestação (primeira: 16–20 semanas; segunda: 28 semanas gestacionais) e três avaliações no pós-parto (aos 3, 6 e 12 meses), nas quais foram coletados dados demográficos e socioeconômicos, gestacionais, de estilo de vida e clínicos. Utilizou-se o *Self-Reported Questionnaire* (escore ≥ 8) para rastreamento do transtorno mental comum gestacional e a escala de depressão pós-natal de Edimburgo (escore ≥ 10) para identificação de sintomas depressivos pós-parto. Foi utilizada regressão logística ordinal ajustada para investigar a relação entre as covariáveis e a ocorrência de transtornos mentais comuns na gravidez e a sintomatologia depressiva no pós-parto.

**RESULTADOS:**

Um total de 461 mulheres completaram as duas avaliações clínicas na gestação; dessas, 247 completaram as três avaliações pós-parto. A ocorrência de transtorno mental comum durante a gestação foi de 36,2% e 24,5% na primeira e segunda avaliações, respectivamente, e a incidência cumulativa foi de 9,2%. Ademais, 50,3% mantiveram o transtorno entre as avaliações. Durante o pós-parto, aproximadamente 20% das mães apresentaram sintomatologia depressiva ao longo do primeiro ano de vida de seus filhos. A paridade (≥ 2) foi associada ao transtorno mental comum, enquanto a baixa escolaridade materna associou-se com sintoma depressivo pós-parto. Mulheres com transtorno mental comum nas duas avaliações na gravidez apresentaram 5,6 vezes mais chance (IC95% 2,50–12,60) de desenvolverem sintoma depressivo pós-parto.

**CONCLUSÃO:**

A ocorrência de transtorno mental comum em qualquer momento avaliado durante a gravidez, mas principalmente sua persistência a partir do segundo trimestre, foi fortemente associado ao sintoma depressivo posterior ao parto. Tais achados evidenciam a necessidade de rastreamento precoce e monitoramento da saúde mental de gestantes no início do pré-natal, a fim de reduzir possíveis impactos negativos para a saúde do binômio mãe-filho causados por tais eventos.

## INTRODUÇÃO

Durante o período perinatal, compreendido entre o início da gestação até os 12 meses pós-parto, as mulheres estão sujeitas a alguns tipos de problemas mentais^1^, dentre os quais o transtorno mental comum (TMC) é um dos mais prevalentes. O TMC é uma suspeita de comorbidade mental para os transtornos de humor, ansiedade e somatização. Suas características envolvem a sintomatologia depressiva e ansiosa, desconcentração, esquecimento, insônia, fadiga, irritabilidade e queixas somáticas não específicas, que podem gerar consternação e disfunção nas atividades diárias^2^. Já no período pós-parto há maior susceptibilidade à ocorrência de sintomas depressivos não psicóticos com humor disfórico, distúrbio psicomotor, alterações no sono e no apetite, fadiga, sentimento de desvalia ou culpa excessiva, pensamentos recorrentes de morte, com ideação suicida, sentimento de inadequação e rejeição ao bebê^2^.

Recentes revisões sistemáticas apontam para as elevadas ocorrências do TMC gestacional no mundo: em países de alta renda estima-se uma prevalência entre 10%–15%, enquanto em países de baixa e média renda a ocorrência fica entre 10%–41%^3 , 4^. A respeito da sintomatologia depressiva no pós-parto, as prevalências estimadas variam entre 24%–27% e entre 14%–50% em países de alta e de baixa e média renda, respectivamente^4^. No Brasil, estudos apontam para alarmantes prevalências de TMC na gestação (63%) e de sintomas depressivos pós-parto (26%)^5^.

Diversos fatores têm sido apontados como potenciais determinantes de tais problemas mentais no período perinatal, como os biológicos, psicológicos e os sociais^6^. Nesse sentido, alguns fatores de risco particularmente são relevantes em algumas culturas e no contexto de países de baixa e média renda, o que ajuda a explicar a grande magnitude dos problemas mentais durante a gestação e pós-parto em certos locais. Durante a gestação, destacam-se a gravidez na adolescência, gravidez não planejada ou indesejada, nuliparidade, baixo nível socioeconômico, alta aglomeração familiar no domicílio e violência física, psicológica e verbal perpetrada pelo companheiro^6 , 7^. No pós-parto, são comumente associados ao adoecimento mental os sintomas depressivos e ansiosos na gestação, história pregressa de transtorno mental, falta de suporte familiar, viver sem companheiro, ser mãe solteira e multiparidade^7 , 8^. Ademais, a ocorrência de TMC na gestação tem sido apontada como um importante preditor para o aparecimento de sintomas depressivos no pós-parto^3^.

A ocorrência de problemas mentais no período perinatal tem profundos impactos negativos tanto para o binômio mãe-filho quanto para toda a família e sociedade. Desfechos obstétricos e neonatais desfavoráveis – como complicações obstétricas e experiências negativas do parto, prematuridade, baixo peso ao nascer e interação mãe-bebê prejudicada – têm sido apontados como consequências dos transtornos mentais no período perinatal, além dos problemas familiares decorrentes do surgimento ou agravamento de conflitos conjugais, por exemplo^6 , 9^.

Apesar da importância e do impacto que os problemas mentais ocorridos no período perinatal têm na vida familiar e social da mulher acometida, a compreensão desse fenômeno entre as mulheres de países de baixa e média renda ainda é limitada e requer investigação para o estabelecimento de estratégias preventivas e promotoras de saúde^3 , 4^.

Este estudo busca investigar a frequência e os fatores associados com os TMC e sintomas depressivos no pós-parto, bem como a associação entre ambos em uma coorte de gestantes atendidas durante o pré-natal na atenção primária à saúde em município da Amazônia Ocidental Brasileira. Hipotetizamos que a ocorrência de TMC gestacional seja um fator de risco para surgimento de sintomatologia depressiva pós-natal.

## MÉTODOS

### Desenho, Local e População de Estudo

Trata-se de uma coorte prospectiva que integra o MINA-Brasil (Saúde e Nutrição Materno-Infantil em Cruzeiro do Sul, Acre)^10^, cujo objetivo é investigar determinantes de saúde e nutrição do binômio mãe-filho desde o período pré-natal até os dois primeiros anos de vida da criança em Cruzeiro do Sul, Acre. O município é o segundo mais populoso do estado com cerca de 82.000 mil habitantes, dos quais 70% vive na zona urbana. Situa-se a 640 km de Rio Branco^11^, capital do estado. O índice de desenvolvimento humano do município em 2010 foi de 0,664^11^. Somente 12,7% dos domicílios tiveram acesso a saneamento adequado em 2010^11 , 12^.

Foram consideradas para participar do estudo mulheres com até 20 semanas de gestação, de acordo com a data da última menstruação, que viviam na área urbana, que realizaram o pré-natal nas 13 unidades de atenção primária à saúde e com intenção de realizar o parto na única maternidade do município. Somente mulheres com gravidez única e de baixo risco obstétrico foram incluídas na presente análise^10 , 13^.

O rastreamento das participantes ocorreu semanalmente entre fevereiro de 2015 e janeiro de 2016. Nesse período, as equipes da Estratégia de Saúde da Família registravam de modo padronizado todos os contatos das potenciais participantes. Posteriormente, foi realizado contato telefônico para explicação do protocolo de pesquisa e o convite para a participação no estudo.

A partir do aceite à participação, a equipe de pesquisa realizou visitas domiciliares para obtenção da assinatura do Termo de Consentimento Livre e Esclarecido (TCLE) e aplicação dos questionários sociodemográficos e de história de saúde^10 , 13^. Em seguida, foi agendada a primeira avaliação que deveria ocorrer entre 16 e 20 semanas gestacionais para a coleta de dados clínicos, amostras de sangue, informações adicionais de história de saúde e estilo de vida, além de realização de ultrassonografia para confirmação da idade gestacional. Uma segunda avaliação clínica ocorreu quando as mulheres estavam com cerca de 28 semanas de idade gestacional. Ambas as avaliações ocorreram entre março de 2015 e maio de 2016^10 , 13^.

Entre julho de 2015 e junho de 2016 as participantes realizaram seus partos na única maternidade do município. No pós-parto, aos 3 meses foi realizada entrevista telefônica entre agosto de 2015 e setembro de 2016 no Laboratório de Pesquisas Telefônicas da Faculdade de Saúde Pública da Universidade de São Paulo. As seguintes avaliações ocorreram aos 6 meses (janeiro e dezembro de 2016) e 12 meses (agosto de 2016 e julho de 2017) após o parto por meio de avaliações presenciais. Em cada uma dessas três avaliações no pós-parto foram coletadas informações sobre a saúde mental materna^10^. Mais detalhes sobre as etapas estão apresentados na Figura .

**Figura:** Fluxograma de recrutamento e dos seguimentos gestacional e pós-natal de mulheres participantes do estudo MINA-Brasil.

### Exposição e Desfecho

Para a presente análise, foram considerados os desfechos de interesse a ocorrência de TMC na primeira e segunda avaliações, realizadas durante a gestação, e de sintomas depressivos no período pós-parto aos 3, 6 e 12 meses.

Para o rastreamento da exposição de interesse caracterizada por sintomas depressivos, ansiosos e algumas queixas psicossomáticas nos últimos 30 dias, foi utilizada a versão brasileira do *Self-Reported Questionnaire* (SRQ-20), validada em 1986 por Mari e Williams^14^, que tem índice de consistência interna (alpha de Cronbach) de 0,86. O SRQ-20 conta com 20 questões com respostas do tipo não ou sim, de modo que cada resposta positiva equivale a um ponto. Quanto maior a frequência de respostas positivas, maior a intensidade de TMC. Adotou-se o ponto de corte ≥ 8 para a classificação de TMC na gravidez (sensibilidade de 75,4% e especificidade de 90,9%)^15^.

Para avaliação do sintoma depressivo no período pós-natal foi utilizada a Escala de Depressão Pós-natal de Edimburgo (EPDS), que estima a ocorrência de sintomas depressivos nos últimos sete dias^16^. A versão utilizada neste estudo foi traduzida e validada por Santos et al.^17^, que obteve um índice de consistência interna (alpha de Cronbach) de 0,87. O questionário é estruturado com dez perguntas e com quatro respostas possíveis cada, sendo que a pontuação em cada questão varia de 0 a 3 e o total, de 0 a 30 pontos. Quanto maior a frequência de respostas positivas, mais intensa a sintomatologia depressiva, sendo adotado o ponto de corte ≥ 10 para a presente análise (sensibilidade de 82,7% e especificidade de 65,3%)^17^.

### Covariáveis

As covariáveis, contínuas ou discretas, foram divididas em três grupos: demográficas e socioeconômicas; gestacionais; estilo de vida e clínicas. As variáveis demográficas e socioeconômicas foram: idade materna em anos (≤ 19, 20–34, ≥ 35), escolaridade materna em anos (0–9, 10–11, ≥ 12), cor da pele autorreferida (branca, preta, parda, indígena ou amarela), morar com companheiro (não/sim), chefe da família (mãe, companheiro, outra pessoa), presença aglomeração familiar (≥ 2 pessoas por cômodo no domicílio, não/sim), recebe Bolsa Família (não/sim), presença de bens de consumo no domicílio.

Para avaliação do nível socioeconômico das participantes do estudo, calculou-se o índice de riqueza baseado na posse de bens de consumo e utilidades domésticas no domicílio. O índice de bens foi obtido a partir da análise de componentes principais, posteriormente dividido em quintis, sendo que o primeiro quintil representa os 20% mais pobres e o quintil mais alto, os 20% mais ricos. A adoção desse método se justifica por refletir melhor a situação econômica familiar do que apenas considerar a renda mensal dos integrantes da família, que é uma informação imprecisa e de difícil obtenção^10^.

As variáveis gestacionais foram: paridade (0, 1, ≥ 2), gravidez planejada (não/sim); estilo de vida: fumo (não/sim) e consumo de álcool (não/sim); avaliação clínica: anemia gestacional (não/sim), Índice de Massa Corporal (IMC) pré-gestacional (baixo peso, eutrofia, sobrepeso, obesidade). A anemia gestacional foi determinada a partir da concentração de hemoglobina (Hb) sanguínea com a utilização hemoglobinômetro portátil da marca Hemocue^®^(Ängelholm, Suécia) com sangue venoso. Para classificar a anemia na gestante adotou-se a classificação de < 110g/L ao nível do mar, recomendada pela Organização Mundial da Saúde (OMS)^18^. O peso pré-gestacional autorreferido pelas gestantes foi obtido no momento da primeira avaliação clínica, considerando o período até 2 meses antes da gestação^19^. A altura das participantes foi mensurada utilizando estadiômetro portátil vertical (altura exata, Belo Horizonte, Brasil) com precisão de 0,1 cm e capacidade para 213 cm. Para classificação antropométrica das gestantes com idade maior ou igual a 19 anos foi utilizado o IMC pré-gestacional, calculado pela divisão do peso pré-gestacional, em kg, pela altura, em metros, elevada ao quadrado (IMC = peso pré-gestacional/altura^2^), e classificado segundo critérios da OMS^19^: baixo peso (IMC < 18,5 kg/m^2^); eutrofia (IMC = 18,5–24,9 kg/m^2^); sobrepeso (IMC = 25,0–29,9 kg/m^2^) e obesidade (≥ 30 kg/m^2^). Já para as gestantes com idade menor do que 19 anos, a definição do estado nutricional foi realizada com auxílio do programa WHO Anthro Plus (http://www.who.int/growthref/tools/en/) que calcula IMC para idade em escore-z, considerando os seguintes pontos de corte para sua classificação: baixo peso (escore-z < -2); eutrofia (escore-z -2 ˫ +1); sobrepeso (escore-z ≥ +1) e obesidade (escore-z ≥ +2).

### Análises Estatísticas

Medidas de tendência central e dispersão foram calculadas e utilizadas na descrição da amostra (média, mediana e desvio-padrão [DP]), bem como os respectivos intervalos com 95% de confiança (IC95%). A partir das frequências de TMC em cada uma das avaliações foi determinada a incidência cumulativa (proporção de gestantes com SRQ-20 positivo na segunda avaliação entre todas aquelas que tiveram um resultado negativo na primeira avaliação) e a persistência do TMC (proporção de mulheres com resultado positivo na segunda avaliação entre todas aquelas com resultado positivo na primeira avaliação) entre a primeira e a segunda avaliação. Verificamos a existência de possíveis diferenças entre as participantes que não foram acompanhadas e aquelas que completaram as avaliações durante a gestação e no pós-parto, pela comparação das médias da idade materna, índice de riqueza e anos de escolaridade utilizando o teste *t* .

Para determinar os fatores associados aos desfechos TMC na gestação e sintoma depressivo no pós-parto, separadamente, adotou-se o modelo de seleção hierarquizado de covariáveis, proposto por Victora et al.^20^Para essa análise, as covariáveis foram agrupadas conforme os seguintes níveis, do mais distante ao mais próximo: socioeconômicas; gestacionais e obstétricas; e clínicas. A análise ajustada levou em consideração o efeito de cada variável controlando para variáveis de confusão no mesmo nível e em níveis superiores. A análise ajustada incluiu apenas as variáveis com um valor de p < 0,20 em cada nível.

Utilizamos a regressão logística ordinal ajustada para investigar a relação entre as covariáveis e a ocorrência de TMC na gravidez e a sintomatologia depressiva no pós-parto. Para isso, as informações coletadas nos diferentes períodos durante a gestação e pós-parto foram combinadas conforme a persistência de cada sintomatologia. Com relação ao TMC, mulheres com SRQ-20 < 8 pontos nas duas avaliações na gravidez foram classificadas como “nunca apresentaram TMC”, mulheres com SRQ-20 ≥ 8 em pelo menos uma avaliação foram classificadas como “alguma vez” e as participantes com SRQ-20 ≥ 8 nas duas avaliações foram classificadas como “sempre apresentaram TMC”. Semelhante abordagem foi adotada para a sintomatologia depressiva no pós-parto, com base na pontuação definida para este estudo (EPDS ≥ 10), que classifica as mulheres apresentando sintomatologia depressiva como “nunca”, “alguma vez” ou “sempre” após o nascimento do bebê. A regressão logística ordinal assume que o efeito de exposição é o mesmo para todas as divisões de categoria da variável de resultado. As análises dos dados foram realizadas com auxílio do pacote estatístico Stata 14.0.

### Aspectos Éticos

O MINA-Brasil foi aprovado pelo Comitê de Ética em Pesquisa da Faculdade de Saúde Pública da Universidade de São Paulo (parecer nº 872.613, 13/11/2014). A presente análise foi aprovada pelo Comitê de Ética em Pesquisa da Universidade Federal de São Paulo (parecer nº 2.726.516, 20/06/2018). A participação no estudo foi voluntária, mediante a assinatura do TCLE.

## RESULTADOS

Foram rastreadas 860 gestantes durante o período de estudo, das quais 699 (81,3%) eram elegíveis para participar no estudo. Dessas, 588 responderam ao questionário socioeconômico. Um total de 528 participantes (75,5% das elegíveis) completaram a primeira avaliação clínica, nas 16ª a 20ª semana gestacional, e 470 (67,2% das elegíveis) completaram a segunda avaliação, na 28^a^semana gestacional, sendo que 461 gestantes atenderam às duas avaliações clínicas. No período pós-parto, 393, 339 e 345 mulheres responderam ao EPDS aos 3, 6 e 12 meses, respectivamente. Foram incluídas na análise longitudinal 247 participantes que responderam ao EPDS em todos os momentos pós-parto ( Figura ).

Na comparação das características socioeconômicas entre as participantes acompanhadas durante todas as etapas do estudo e as perdas de seguimento, não encontramos diferenças entre as médias de idade (TMC: 24,9 e 23,9 anos; p = 0,152; Sintoma depressivo: 25,2 e 24,3 anos; p = 0,076) e anos de escolaridade materna (TMC: 10,5 e 9,9 anos; p = 0,055; Sintoma depressivo: 10,6 e 10,2 anos; p = 0,076). Encontramos diferenças apenas para o índice de riqueza, em que as mulheres que participaram das avaliações clínicas tanto na gestação quanto no pós-parto apresentaram uma maior média do índice em comparação àquelas que não completaram as avaliações (TMC: 1,8 e 1,6; p = 0,001; Sintoma depressivo: 1,9 e 1,7; p = 0,008).

A ocorrência de TMC na primeira e segunda avaliação na gestação foi de 36,2% (IC95% 32,1–40,4) e 24,5% (IC95% 20,6–28,6), respectivamente. A incidência cumulativa de TMC foi de 9,2% (IC95% 6,1–13,1) e a persistência do sintoma ocorreu em metade das gestantes, 50,3% (IC95% 42,5–58,1). No período pós-natal observou-se prevalências de 22% (IC95% 18,6–27,1) de sintoma depressivo aos 3 meses, 20,9% (IC95% 16,7–25,7) aos 6 meses e 20,6% (IC95% 16,4–25,2) aos 12 meses pós-parto (dados não apresentados em tabela).

A Tabela 1 apresenta as características socioeconômicas, gestacionais e obstétricas e clínicas das participantes. A maioria das participantes tinham entre 20–34 anos (65%), cerca de 30% tinham menos de 9 anos de escolaridade e 40% eram beneficiárias do Bolsa Família. Menos de 15% das mulheres eram chefes de seus domicílios, a grande maioria residia com um companheiro (77,6%) e 44% eram primigestas ( Tabela 1 ). A idade gestacional média na primeira avaliação foi de 20 semanas (DP = 2,91) e de 27,7 semanas (DP = 1,61) na segunda avaliação (dados não apresentados em tabelas).

**Tabela 1:** Características das participantes e prevalência, incidência e persistência de transtornos mentais comuns na gestação. Cruzeiro do Sul, Acre, 2015–2017.

Variáveis	16ª a 20ª semana gestacional			28^a^semana gestacional			Incidência		Persistência		Transtornos mentais comuns entre a 1ª e 2ª avaliação				
				
	n (%)	Prevalência (%)	p	n (%)	Prevalência (%)	p	%	p	%	p	n (%)	Nunca	Alguma vez	Sempre	p
Índice de riqueza (quintis)			0,201			0,263		0,817		0,163					0,227
1	95 (18,0)	41,1		79 (16,8)	29,1		4,4		61,3		76 (16,5)	56,6	18,4	25,0	
2	110 (20,9)	42,7		95 (20,2)	30,5		9,1		59,0		94 (20,4)	53,2	22,3	24,5	
3	106 (20,1)	34,9		98 (20,9)	24,5		9,7		52,9		96 (20,8)	58,3	22,9	18,8	
4	109 (20,7)	33,0		101 (21,5)	19,8		10,8		38,2		99 (21,5)	58,6	28,3	13,1	
5	107 (20,3)	29,0		97 (20,6)	19,6		10,5		37,9		96 (20,8)	62,5	26,0	11,5	
Chefe da família			0,565			0,075		0,313		0,107					0,103
Mãe	72 (13,7)	41,7		65 (13,8)	35,4		10,8		66,7		64 (13,9)	51,6	20,3	28,1	
Companheiro	278 (52,8)	35,3		248 (52,8)	21,8		6,7		50,6		245 (53,1)	60,8	21,6	17,6	
Outro	177 (33,6)	35,0		157 (33,4)	24,2		12,4		41,8		152 (33,0)	55,9	29,0	15,1	
Recebe Bolsa Família			0,588			0,154		0,227		0,574					0,356
Não	316 (60,0)	35,1		288 (61,3)	22,2		7,6		48,5		281 (61,0)	60,5	22,8	16,7	
Sim	211 (40,0)	37,4		182 (38,7)	28,0		11,8		52,9		180 (39,0)	53,9	25,6	20,6	
Presença de aglomeração familiar (≥ 2 pessoas por cômodo)			0,078			0,069		0,988		0,275					0,047
Não	469 (89,0)	34,8		425 (90,4)	23,3		9,2		48,6		416 (90,2)	59,4	23,8	16,8	
Sim	58 (11,0)	46,6		45 (9,6)	35,6		9,1		60,9		45 (9,8)	44,4	24,4	31,1	
Escolaridade materna (anos)			0,087			0,107		0,412		0,580					0,329
0–9	142 (27,9)	42,3		126 (27,8)	28,6		8,6		52,9		121 (27,1)	52,9	24,8	22,3	
10–11	242 (47,5)	34,3		213 (46,9)	25,8		11,6		52,8		210 (47,1)	58,1	23,8	18,1	
≥ 12	125 (24,6)	29,6		115 (25,3)	17,4		6,3		42,9		115 (25,8)	65,2	21,7	13,0	
Idade materna (anos)			0,450			0,951		0,241		0,352					0,418
≤ 19	143 (27,1)	32,2		123 (26,2)	23,6		13,6		40,5		118 (25,6)	59,3	28,0	12,7	
20–34	343 (65,1)	37,0		309 (65,7)	24,9		7,9		53,9		306 (66,4)	57,5	22,2	20,3	
≥ 35	41 (7,8)	41,5		38 (8,1)	23,7		4,6		46,7		37 (8,0)	56,8	24,3	18,9	
Mora com companheiro			0,160			0,263		0,501		0,393					0,345
Não	118 (22,4)	41,5		101 (21,5)	28,7		6,9		56,1		99 (21,5)	54,6	22,2	23,2	
Sim	409 (77,6)	34,5		369 (78,5)	23,3		9,8		48,4		361 (78,5)	58,8	24,3	16,9	
Cor da pele			0,549			0,639		0,826		0,571					0,655
Branca	76 (14,4)	38,2		70 (14,9)	20,0		4,8		42,9		70 (15,1)	57,1	25,7	17,1	
Preta	23 (4,4)	39,1		20 (4,3)	30,0		7,7		71,4		20 (4,3)	60,0	15,0	25,0	
Parda	405 (76,9)	35,1		363 (77,2)	24,8		10,0		51,2		354 (76,8)	58,8	23,5	17,8	
Indígena	4 (0,8)	75,0		4 (0,9)	50,0		0		66,7		4 (0,9)	25,0	25,0	50,0	
Amarela	19 (3,6)	36,8		13 (2,8)	23,1		14,3		33,3		13 (2,8)	46,2	38,5	15,4	
Planejou a gravidez			0,007			0,283		0,913		0,921					0,097
Não	291 (55,3)	41,2		257 (54,8)	26,5		9,4		50,0		253 (55,0)	53,4	26,1	20,6	
Sim	235 (44,7)	29,8		212 (45,2)	22,2		9,1		50,8		207 (45,0)	63,3	21,3	15,5	
Paridade			< 0,001			0,027		0,674		0,205					0,005
0	234 (44,4)	29,1		210 (44,7)	22,9		10,6		49,2		202 (43,8)	62,4	22,8	14,9	
1	134 (25,4)	31,3		120 (25,5)	18,3		8,5		39,5		120 (26,0)	62,5	25,0	12,5	
≥ 2	159 (30,2)	50,3		140 (29,8)	32,1		7,0		57,4		139 (30,2)	47,5	24,5	28,1	
IMC pré-gestacional			0,482			0,905		0,822		0,539					0,626
Baixo peso	38 (7,2)	44,7		35 (7,5)	28,6		5,0		69,2		33 (7,2)	57,6	15,2	27,3	
Eutrofia	325 (61,6)	35,4		287 (61,1)	24,4		10,0		48,1		284 (61,6)	57,0	25,4	17,6	
Sobrepeso	128 (24,2)	33,6		114 (24,3)	22,8		9,3		48,6		110 (23,9)	61,8	22,7	15,5	
Obesidade	37 (7,0)	43,2		34 (7,2)	26,5		5,3		53,3		34 (7,4)	52,9	23,5	23,5	
Anemia gestacional na 1ª avaliação			0,965			0,547		0,482		0,128					0,248
Não	437 (84,9)	36,2		384 (84,4)	23,4		9,8		47,5		384 (84,4)	57,6	25,3	17,2	
Sim	78 (15,1)	35,9		71 (15,6)	26,8		6,5		64,0		71 (15,6)	60,6	16,9	22,5	
Fumo na 1ª avaliação			0,006			0,024		0,431		0,124					0,007
Não	508 (96,2)	35,0		444 (96,3)	23,2		0		48,7		444 (96,3)	58,8	24,1	17,1	
Sim	20 (3,8)	65,0		17 (3,7)	47,1		9,4		72,7		17 (3,7)	35,3	17,7	47,1	
Consumo de álcool na 1ª avaliação			0,003			0,060		0,206		0,161					0,010
Não	486 (92,1)	34,4		426 (92,4)	23,0		9,7		48,3		426 (92,4)	59,2	24,2	16,7	
Sim	42 (7,9)	57,1		35 (7,6)	37,1		0		65,0		35 (7,6)	42,9	20,0	37,1	

IMC: índice de massa corporal.

Na análise bruta, apresentaram com maior frequência TMC “sempre” durante a gravidez: mulheres de famílias em que existia aglomeração, aquelas com paridade ≥ 2 e aquelas que fumaram e/ou que consumiram álcool durante a gravidez ( Tabela 1 ). A Tabela 2 apresenta o modelo final ajustado dos fatores associados ao TMC em algum momento da gravidez. Apenas a paridade ≥ 2 esteve significativamente associada ao desfecho, com uma razão de chances de 1,72 (IC95% 1,12–2,66).

**Tabela 2:** Fatores associados à ocorrência de transtorno mental comum entre a primeira e segunda avaliações clínicas na gestação. Cruzeiro do Sul, Acre, 2015–2017.

Variáveis	OR_bruto_(IC95%)	OR_ajustado_(IC95%)	p
Presença de aglomeração familiar (≥ 2 pessoas por cômodo)			0,109
Não	Referência	Referência	
Sim	1,49 (0,86–2,57)	1,62 (0,87–2,94)	
Planejou a gravidez			0,101
Não	1,56 (1,10–2,21)	1,35 (0,93–1,96)	
Sim	Referência	Referência	
Paridade			0,013
0	Referência	Referência	
1	1,05 (0,67–1,62)	0,96 (0,61–1,53)	
≥ 2	2,11 (1,40–3,17)	1,72 (1,12–2,66)	
Fumo na 1ª avaliação			0,144
Não	Referência	Referência	
Sim	2,74 (1,08–7,00)	2,06 (0,78–5,47)	
Consumo de álcool na 1ª avaliação			0,052
Não	Referência	Referência	
Sim	2,01 (1,06–3,80)	1,95 (0,99–3,83)	

OR: *odds ratio* ; IC95%: intervalo de confiança de 95%.

Na Tabela 3 está apresentada a análise bruta para a ocorrência de sintomatologia depressiva no pós-parto. Apresentaram com maior frequência sintomatologia depressiva “sempre” durante a gravidez: mulheres de famílias com menor índice de riqueza, aquelas em que a mãe era chefe da família, mulheres de menor escolaridade, com paridade ≥ 2, que apresentaram anemia gestacional na primeira avaliação e aquelas que consumiram álcool durante a gravidez ( Tabela 3 ). Mulheres que apresentaram SRQ positivo tanto na primeira como na segunda avaliação apresentaram as maiores frequências de sintomatologia depressiva no pós-parto. Na análise ajustada para potenciais fatores de confusão, mulheres menos escolarizadas e aquelas com história de TMC durante a gravidez apresentaram as maiores chances de sintomatologia depressiva no pós-parto ( Tabela 4 ). Ter menos de 12 anos de escolaridade elevou as chances de depressão pós-parto em mais de três vezes, comparativamente às mulheres com 12 ou mais anos de escolaridade. As gestantes que relataram sempre ter apresentado TMC durante a gestação tiveram 5,6 vezes maiores chances (IC95% 2,50–12,60) de apresentar sintomatologia depressiva pós-parto em comparação àquelas que nunca relataram o problema ( Tabela 4 ).

**Tabela 3:** Variáveis associadas à ocorrência de sintomatologia depressiva entre 3, 6 e 12 meses pós-natal. Cruzeiro do Sul, Acre, 2015–2017 (n = 247).

Variáveis	n (%)	Sintomatologia depressiva entre os 3, 6 e 12 meses			p

		Nunca	Alguma vez	Sempre	
Índice de riqueza (quintis)					0,001
1	35 (14,2)	45,7	25,7	28,6	
2	53 (21,5)	62,3	30,2	7,6	
3	47 (19,0)	61,7	27,7	10,6	
4	59 (23,9)	69,5	28,8	1,7	
5	53 (21,5)	79,3	17,0	3,8	
Chefe da família					0,040
Mãe	29 (11,7)	58,6	17,2	24,1	
Companheiro	139 (56,3)	64,8	28,1	7,2	
Outra pessoa	79 (32,0)	68,4	25,3	6,3	
Recebe Bolsa Família					0,197
Não	153 (61,9)	69,3	23,5	7,2	
Sim	94 (38,1)	58,5	29,8	11,7	
Presença de aglomeração familiar (≥ 2 pessoas por cômodo)					0,057
Não	237 (96,0)	65,8	26,2	8,0	
Sim	10 (4,1)	50,0	20,0	30,0	
Escolaridade materna (anos)					0,002
0–9	54 (22,8)	53,7	31,5	14,8	
10–11	118 (49,8)	59,3	31,4	9,3	
≥ 12	65 (27,4)	84,6	13,9	1,5	
Idade materna (anos)					0,720
≤ 19	62 (25,1)	72,6	21,0	6,5	
20–34	167 (67,6)	62,9	27,5	9,6	
≥ 35	18 (7,3)	61,1	27,8	11,1	
Mora com companheiro					0,123
Não	54 (21,9)	66,7	18,5	14,8	
Sim	193 (78,1)	64,8	28,0	7,3	
Cor da pele					0,443
Branca	30 (12,2)	60,0	23,3	16,7	
Preta	11 (4,5)	36,7	45,5	18,2	
Parda	195 (79,0)	67,2	25,1	7,7	
Indígena	4 (1,6)	75,0	25,0	0	
Amarela	7 (2,8)	71,4	28,6	0	
Planejou a gravidez					0,155
Não	133 (53,9)	61,7	26,3	12,0	
Sim	114 (46,2)	69,3	25,4	5,3	
Paridade					0,044
0	116 (47,0)	70,7	23,3	6,0	
1	59 (23,9)	71,2	22,0	6,8	
≥ 2	72 (29,1)	51,4	33,3	15,3	
IMC pré-gestacional					0,259
Baixo peso	18 (7,6)	44,4	33,3	22,2	
Eutrofia	140 (58,8)	69,3	24,3	6,4	
Sobrepeso	66 (27,7)	60,6	30,3	9,1	
Obesidade	14 (5,9)	71,4	21,4	7,1	
Anemia gestacional na 1ª avaliação					0,026
Não	200 (81,0)	69,0	22,5	8,5	
Sim	47 (19,0)	48,9	40,4	10,6	
Fumo na 1ª avaliação					0,391
Não	237 (96,0)	65,8	25,7	8,4	
Sim	10 (4,0)	50,0	30,0	20,0	
Consumo de álcool na 1ª avaliação					0,004
Não	226 (91,5)	66,8	26,1	7,1	
Sim	21 (8,5)	47,6	23,8	28,6	
SRQ positivo na 1ª avaliação					< 0,001
Não	157 (66,5)	73,9	24,2	1,9	
Sim	79 (33,5)	46,8	31,7	21,5	
SRQ positivo na 2ª avaliação					< 0,001
Não	168 (76,4)	70,8	25,0	4,2	
Sim	52 (23,6)	44,2	34,6	21,2	
SRQ positivo em algum momento durante a gravidez					< 0,001
Nunca	130 (59,6)	73,1	24,6	2,3	
Em uma avaliação	49 (22,5)	63,3	28,6	8,2	
Sempre	39 (17,9)	35,9	35,9	28,2	

IMC: índice de massa corporal; SRQ: *Self-Reported Questionnaire* .

**Tabela 4:** Fatores associados à sintomatologia depressiva entre 3, 6 e 12 meses pós-natal. Cruzeiro do Sul, Acre, 2015–2017.

Variáveis	OR_bruto_(IC95%)	OR_ajustado_(IC95%)	p
Índice de riqueza (quintis)			0,200
1	5,87 (2,30–14,95)	3,09 (1,15–8,31)	
2	2,27 (0,96–5,33)	1,56 (0,62–3,92)	
3	2,43 (1,01–5,84)	1,96 (0,79–4,90)	
4	1,57 (0,67–3,69)	1,33 (0,55–3,23)	
5	Referência	Referência	
Escolaridade materna (anos)			0,007
0–9	5,29 (1,83–15,27)	3,89 (1,58–9,62)	
10–11	3,56 (1,31– 9,66)	3,16 (1,44–6,93)	
≥ 12	Referência	Referência	
Anemia na gestação			0,075
Não	Referência	Referência	
Sim	0,48 (0,26–0,88)	1,87 (0,94–3,72)	
Consumo de álcool na gestação			0,081
Não	Referência	Referência	
Sim	2,77 (1,09–7,01)	2,61 (0,89–7,65)	
SRQ positivo em algum momento durante a gravidez			< 0,001
Nunca	Referência	Referência	
Em uma avaliação	1,65 (0,83–3,29)	1,87 (0,90–3,92)	
Sempre	6,19 (2,95–12, 97)	5,6 (2,50–12,60)	

OR: *odds ratio* ; IC95%: intervalo de confiança de 95%; SRQ: *Self-Reported Questionnaire.*

## DISCUSSÃO

Neste estudo prospectivo com gestantes que realizaram o cuidado pré-natal na atenção primária à saúde em município da Amazônia Ocidental Brasileira, o TMC gestacional ocorreu em mais de 35% das gestantes no segundo trimestre e em cerca de um quarto das participantes no terceiro trimestre gestacional, sendo que aproximadamente 20% das mães apresentaram sintomatologia depressiva pós-parto ao longo do primeiro ano de vida de seus filhos. Foi observado também que mulheres que apresentaram TMC no segundo e terceiro trimestres tiveram quase seis vezes maiores chances de desenvolver sintomas depressivos pós-parto em comparação às mulheres que não reportaram TMC gestacional. Mulheres com dois ou mais filhos apresentaram maiores chances de sofrer TMC na gravidez e aquelas com menor escolaridade apresentaram maiores chances de apresentar sintomatologia depressiva no pós-natal.

A frequência de TMC em diferentes momentos na gestação em nosso estudo foi superior àquela descrita em uma meta-análise que incluiu mais de 5.700 participantes de países de baixa e média renda, 15,6% (IC95% 15,4–15,9), bem como em estudo observacional conduzido na Etiópia (26,2%) rastreado com o SRQ-20 escore ≥ 6^3 , 21^. No Brasil, um estudo transversal encontrou elevada ocorrência de TMC no segundo trimestre de gravidez (51,7%; IC95% 51,7–62,6)^22^, enquanto uma prevalência de 43,1% (IC95% 40,2–46,1) de TMC no terceiro trimestre foi descrita em Pernambuco^23^. Utilizaram o SRQ-20 nos dois estudos com escores ≥ 7 e ≥ 8, respectivamente.

Diferentes estimativas de sintomatologia depressiva no pós-parto também foram observadas em relação aos nossos achados. Revisão sistemática incluindo mais de 38.000 mulheres no pós-parto encontrou prevalência de 19,7% (IC95% 16,9–22,8) de depressão^4^. Em uma coorte incluindo 378 mulheres vietnamitas, os autores descreveram redução na prevalência de sintomatologia depressiva pós-parto (~ 12% no quarto e sexto mês) em comparação ao período gestacional (40% e 28% no primeiro e terceiro trimestre gestacional, respectivamente)^24^. No estudo de base populacional hospitalar “Nascer no Brasil” foi descrita prevalência de 26% de depressão materna, utilizando o EPDS com escore ≥ 13, entre 6 horas e 18 meses após o parto no país; porém, a depressão materna foi mais acentuada entre mulheres da região Norte (31,7%)^25^.

Vários fatores influenciam nas diferentes estimativas para os transtornos mentais ocorridos durante a gestação e pós-parto em todo o mundo. No entanto, os critérios adotados para definir um caso do problema estão entre os que mais influenciam nas discrepâncias encontradas. Apesar de os instrumentos mais utilizados para rastreamento desses problemas no Brasil e na literatura internacional serem o SRQ-20 e o EPDS, os pontos de corte adotados divergiram significativamente entre os estudos, o que explica a variabilidade entre nossos resultados e a literatura sobre o tema^5^. Ademais, é possível verificar a adoção de diferentes conceitos de TMC em alguns estudos, diferentes daqueles estabelecidos pela OMS^2 , 5^.

Diversos fatores econômicos e sociais têm sido associados com a ocorrência de transtornos mentais no período perinatal, dentre os quais a baixa escolaridade materna e as condições socioeconômicas precárias estão entre os mais comumente citados^2 , 8 , 26^. Estudos transversais conduzidos em países de baixa e média renda apontam que a falta de suporte social pela família, principalmente a falta do companheiro durante a gestação, são importantes fatores associados à ocorrência de TMC gestacional, bem como a gravidez não ter sido planejada^21^. Muitos desses fatores de risco ao adoecimento mental na gestação também influenciam em sua ocorrência no pós-parto. Diversos estudos prospectivos e transversais conduzidos em diferentes países apontam que a gestação na adolescência, não planejada, o histórico de doenças mentais pregresso, a falta de suporte social na gestação e os episódios de violência física, sexual e/ou psicológica perpetrada pelo companheiro são fortemente associados aos sintomas depressivos durante o pós-parto^24 , 25 , 27^.

A ocorrência de transtornos mentais durante a gestação tem sido investigada como um importante fator de risco para o adoecimento mental pós-parto. Como demonstram achados de coorte canadense, com 3.057 participantes, a ocorrência de ao menos um fator de risco para saúde mental pré-natal prejudicada (histórico de tratamento de depressão, sintomatologia depressiva e ansiosa pré-natais e experiências psicológicas adversas durante a infância) elevou o risco de depressão materna aos quatro meses (EPDS ≥ 10) em 2,52 vezes, enquanto entre as mulheres que relataram todos os fatores adversos, o risco foi elevado em 7,64 vezes^25^.

A vulnerabilidade mental e emocional pré-natal tende a persistir após o nascimento da criança, ocasionando a transferência dos sintomas já presentes durante a gravidez para o período em que a criança necessita de grande atenção e cuidados maternos. A literatura aponta que tais transtornos perinatais em diferentes momentos compartilham muitos dos fatores de risco associados à sua ocorrência, fatores que tendem a perdurar durante toda essa fase, possibilitando a persistência do acometimento e instabilidade mental durante o curso da gestação e o pós-parto^8 , 25^.

Os nossos achados revelam a importância da detecção precoce de transtornos mentais durante a gravidez para que o cuidado adequado possa ser administrado oportunamente, reduzindo os riscos de continuidade de tais sintomas após o nascimento da criança e os possíveis eventos adversos à saúde associados^6 , 9^. O SRQ-20 e o EPDS são valiosos instrumentos de rápida e fácil aplicação e interpretação, com baixo custo e que dispensam a necessidade de especialista para aplicação. Esses instrumentos podem ser incorporados na rotina da atenção primária à saúde desde o rastreamento precoce de tais condições ainda no pré-natal até o pós-parto^16 , 17^.

Os pontos fortes de nossa análise incluem seu desenho longitudinal com acompanhamento das participantes desde o período pré-natal até 12 meses após o parto, a utilização de métodos validados no Brasil para rastreamento de transtornos mentais no período perinatal e a confirmação da idade gestacional por meio de exames de ultrassom. Dentre as limitações estão as perdas de seguimento durante o acompanhamento, principalmente devido às dificuldades de localização das participantes causadas por restrições comuns na área de estudo, como conexão à internet instável, falta de sinalização adequada nas ruas e o sinal de celular intermitente. A possibilidade de fatores confundidores desconhecidos não terem sido considerados é importante diante da natureza observacional de nosso estudo, como ter sofrido abuso na vida (violência perpetrada por parceiro íntimo, violência sexual, abuso físico ou sexual na infância), pressão para ter um filho e insegurança alimentar.

Entre mulheres atendidas durante o pré-natal na atenção primária à saúde em Cruzeiro do Sul, Acre, encontramos que a ocorrência de TMC é comum durante o segundo e o terceiro trimestres gestacionais e que uma entre cinco mulheres relataram sintomas depressivos pós-natais. A ocorrência de TMC em qualquer dos momentos avaliados durante a gravidez, mas principalmente sua persistência a partir do segundo trimestre, foi fortemente associada ao adoecimento mental posterior ao parto. Esses achados evidenciam a necessidade de rastreamento e monitoramento da saúde mental dessas gestantes por meio da inclusão da avaliação de tais transtornos precocemente, ainda no pré-natal, a fim de reduzir possíveis impactos negativos para a saúde do binômio mãe-filho causados por tais eventos.
